# Evaluation of Maternal and Neonatal Risk Factors in the Transmission of Toxoplasmosis: A Study on the Detection of Toxoplasma gondii Antibodies in Cord Blood

**DOI:** 10.4314/ejhs.v35i4.6

**Published:** 2025-07

**Authors:** Onosakponome Evelyn Orevaoghene, Clement Ugochukwu Nyenke, Roseanne Adah Ikpeama, Wagwu Victoria Chioma

**Affiliations:** 1 Department of Medical Laboratory Science, PAMO University of Medical Sciences, Port Harcourt, Nigeria

**Keywords:** Toxoplasmosis, cord blood, Toxoplasma gondii, maternal factors, neonatal factors

## Abstract

**Background:**

Toxoplasmosis, a neglected tropical disease caused by the parasite Toxoplasma gondii, is among the most common congenital infections worldwide, particularly in developing countries. The infection can be transmitted through cord blood—the blood remaining in the placenta after childbirth. This study investigates the presence of Toxoplasma gondii in cord blood and examines associated maternal and neonatal factors.

**Methods:**

This study was conducted at the Rivers State Teaching Hospital in Port Harcourt. A total of 180 cord blood samples were randomly collected from newborns in the labor ward, following ethical approval. The samples were analyzed for Toxoplasma gondii IgG antibodies using enzyme-linked immunosorbent assay (ELISA). Maternal and neonatal data were collected through structured questionnaires, and statistical analysis was performed using the Statistical Package for the Social Sciences (SPSS).

**Results:**

The findings revealed that 36.7% of the cord blood samples tested positive for Toxoplasma gondii. Maternal age, education level, and occupation were not significantly associated with infection prevalence. However, specific maternal risk factors—including lack of awareness, consumption of undercooked meat, poor hand hygiene, and consumption of unwashed fruits and vegetables—were associated with increased prevalence. Notably, a significant correlation was observed between low birth weight (1.6–2.5 kg) and higher infection rates, with a 25% prevalence in this subgroup.

**Conclusion:**

The high prevalence of toxoplasmosis identified in this study emphasizes the need for increased public health education and awareness regarding the disease and its implications for newborns. Routine screening and appropriate treatment for pregnant women are recommended to reduce the rate of congenital transmission.

## Introduction

Cord blood—the blood remaining in the placenta after delivery—contains a high concentration of stem cells and may harbor congenital pathogens. These pathogens are responsible for congenital infections, which can affect the fetus or newborn and are transmitted vertically from mother to child during pregnancy, childbirth, or breastfeeding. Examples include malaria, toxoplasmosis, trypanosomiasis, syphilis, hepatitis B, rubella, herpes simplex virus, Chagas disease, and human immunodeficiency virus (HIV) (1,2). The overall prevalence of these congenital infections is estimated to be between 0.1 and 0.3 per 1,000 live births (2,3).

Toxoplasmosis, a neglected tropical disease caused by the protozoan Toxoplasma gondii, has been associated with spontaneous abortion in pregnant women and congenital chorioretinitis in neonates (4,5). Vertical transmission from mother to fetus can result in perinatal death or severe developmental abnormalities, depending on the gestational age at the time of infection (5,6). Approximately one-third of maternal infections during pregnancy result in fetal transmission. The tachyzoite, an invasive form of T. gondii, can cross the placental barrier and cause severe congenital toxoplasmosis, often affecting the nervous and visual systems (3,6,7). While the likelihood of transmission increases with gestational age, the severity of fetal damage is typically greater when infection occurs during the first or second trimester (3,6,7).

The global prevalence of toxoplasmosis varies by region. Maternal-fetal transmission rates rise with gestational age—from under 15% at 13 weeks to over 70% at 36 weeks. An estimated 1.2 million cases of congenital toxoplasmosis occur worldwide each year (7,8). In Africa, the pooled prevalence among pregnant women is estimated at 51.01% (9). In Nigeria, reported prevalence rates range widely, from 2% to 88.24% (9). Although toxoplasmosis is one of the most economically and medically significant parasitic infections, it remains underdiagnosed and poorly managed in many parts of sub-Saharan Africa due to a lack of comprehensive data (10).

Given its obstetric and neonatal implications, the early detection of primary toxoplasmosis during pregnancy is essential. This study aims to determine the prevalence of Toxoplasma gondii in cord blood at Rivers State University Teaching Hospital, Port Harcourt.

## Materials and Methods

**Study area**: This study was conducted at Rivers State University Teaching Hospital (RSUTH), a government-owned tertiary healthcare facility located at latitude 4°46′49″N and longitude 7°0′50″E in Port Harcourt Local Government Area, Nigeria. RSUTH is one of the largest hospitals in the Niger Delta and serves as a referral center for primary and secondary health facilities within and beyond Rivers State.

**Study design**: A qualitative cross-sectional study was conducted. A total of 180 cord blood samples were randomly collected from the labor and surgical wards at RSUTH.

**Data collection**: Structured questionnaires were used to collect information on socio-demographic characteristics (age, marital status, occupation, education level) and risk factors related to toxoplasmosis. These included awareness and knowledge of the disease, history of contact with cats, consumption of undercooked meat, eating unwashed fruits and vegetables, drinking untreated water, and handwashing practices. Trained personnel administered the questionnaires to minimize interviewer bias. The questionnaires were pre-tested for clarity and reliability.

**Sample collection and analysis**: Cord blood samples were obtained from the placenta post-delivery in the labor and surgical wards, including after cesarean sections. Five milliliters of cord blood were collected using aseptic techniques into EDTA bottles to prevent contamination. Samples were centrifuged at 1,500 rpm for 5 minutes, and serum was extracted and stored at 2°C. Prior to analysis, all reagents, plates, and specimens were brought to room temperature (24°C).

Samples were tested using the BioCheck Toxoplasma IgG ELISA kit, following the manufacturer's protocol. Results were interpreted as follows: <1.6 = non-reactive (negative), 1.6–3.0 = gray zone, and ≥3.0 = reactive (positive). Each sample was tested in duplicate to ensure reproducibility. Positive and negative controls were included in every assay run to validate results and monitor potential procedural errors (3,11).

**Statistical analysis**: Data were entered into Microsoft Excel and analyzed using SPSS version 25. Descriptive statistics, including frequencies and percentages, were used to summarize participant characteristics and determine the prevalence of Toxoplasma gondii. Chi-square tests were used to assess associations between categorical variables, while Kappa (κ) statistics were applied to evaluate agreement levels between related variables.

**Ethical consideration**: Ethical approval for the study was obtained from the RSUTH Research and Ethics Committee (RSUTH/REC/2022271). In addition, verbal consent was secured from all participants in accordance with ethical guidelines and confidentiality protocols.

## Results

**Figure 1:**
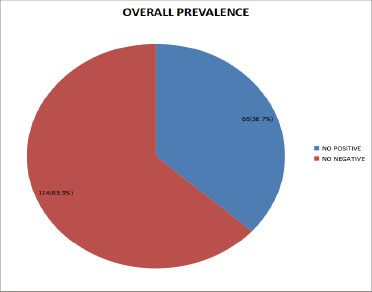
Overall prevalence of toxoplasmosis in cord blood

**Overall prevalence of toxoplasmosis in cord blood**: Figure 1 presents the overall prevalence of Toxoplasma gondii infection in cord blood samples collected in Port Harcourt. A total of 180 cord blood specimens were obtained from mothers during delivery at Rivers State University Teaching Hospital (RSUTH). Among these, 66 samples tested positive for T. gondii, resulting in a prevalence of 36.7%, while 114 samples (63.3%) tested negative. Pearson's Chi-square test revealed a statistically significant association between T. gondii prevalence and cord blood samples (P = 0.000), indicating a meaningful burden of congenital toxoplasmosis in this population.

**Maternal sociodemographic factors and cord blood toxoplasmosis**: Table 1 explores the relationship between maternal sociodemographic variables and the prevalence of toxoplasmosis in cord blood. The highest number of infections (n = 34) occurred among mothers aged 26–35 years, representing a prevalence of 18.9% in this age group. However, statistical analysis using Pearson's Chi-square test showed no significant association between maternal age and T. gondii infection (P = 0.845), suggesting that maternal age did not influence the likelihood of transmission.

**Table 1:** Relationship of maternal sociodemographic factors and toxoplasmosis in cord blood

Factors	Number examined (%)	Positive for *T.gondii* N (%)	P-value
**Age**			
16-25 years	28	9 (5)	0.845
26-35 years	89	34 (18.9)	0.845
36-45 years	63	23 (12.8)	0.845
**Educational status**			
None	32	7 (3.9)	0.298
Primary	28	11 (6.1)	0.298
Secondary	83	33 (18.3)	0.298
Tertiary	37	15 (8.3)	0.298
**Occupation**			
Unemployed	37	11 (6.1)	0.334
Self-employed	94	31 (17.2)	0.334
Employed	32	16 (8.9)	0.334
Retired	4	2 (1.1)	0.334
Student	13	6 (3.3)	0.334
**Overall**	180	66 (36.7)	

P-value – <0.05 is significant Df—degree of freedom

**Neonatal factors and cord blood toxoplasmosis**: Table 2 shows the relationship between neonatal characteristics (sex and birth weight) and T. gondii infection. Female neonates had a slightly higher prevalence (17.2%) compared to males, but this difference was not statistically significant (P = 0.951). In contrast, a significant association was observed between low birth weight (1.6–2.5 kg) and T. gondii positivity. Neonates within this weight range had a prevalence of 25%, and the Chi-square test confirmed this association as statistically significant (P = 0.005).

**Table 2:** Relationship between neonatal factors and toxoplasmosis in cord blood

	Number examined (%)	Number positive (%) *T.gondii*	*p-value*
**Sex**			
Male	96	25 (13.9)	0.951
Female	84	31 (17.2)	0.951
**Birth weight**			
1.6-2.5kg	92	45 (25.0)	0.005
2.6-3.5kg	66	17 (9.4)	0.005
3.6-4.5kg	19	6 (3.3)	0.005
4.6-5.5kg	3	0 (0.0)	0.005
**Total**	180	66 (67.7)	1.922

**Awareness of Toxoplasmosis as a Risk Factor**: Table 3 presents the relationship between maternal awareness of toxoplasmosis and its prevalence in cord blood. The highest infection rate was found among mothers who had no prior knowledge of the disease. Statistical analysis demonstrated a significant association between lack of awareness and increased prevalence of cord blood toxoplasmosis (P = 0.05), indicating that awareness plays a protective role.

**Table 3:** Prevalence of toxoplasmosis in cord blood in relation to awareness as a risk factor

Variables	Responses	Number Examined (%)	Number Positive (%)	*p-value*
Have you heard of Toxoplasmosis	Yes	9(5.0)	3(1.7)	
	No	171(95.5)	63(35.0)	<0.001
Are you aware of congenital toxoplasmosis	Yes	5(2.8)	2(1.1)	
	No	175(97.2)	64(35.6)	<0.001
Do you know Toxoplasmosis can be gotten from pets	Yes	8(4.4)	3(1.7)	
	No	172(95.6)	63(35.0)	<0.001
Are you aware Toxoplasmosis can affect your newborn	Yes	4(2.2)	2(1.1)	
	No	176(97.8)	64(35.6)	<0.001
Have you been Tested of Toxoplasmosis	Yes	-	-	
	No	180(100.0)	66(36.7))	-
		180	66(36.7)	

P-value – <0.05 is significant

**Maternal lifestyle factors and toxoplasmosis prevalence**: Table 4 assesses maternal lifestyle-related risk factors in relation to toxoplasmosis in cord blood. Similar to the findings on awareness, the highest prevalence was recorded among mothers who lacked knowledge of toxoplasmosis. Pearson's Chi-square test again revealed a statistically significant association between maternal lifestyle practices (e.g., hygiene, diet) and infection rates (P = 0.05). This underscores the impact of behavioral risk factors on the likelihood of T. gondii transmission.

**Table 4:** Prevalence of Toxoplasmosis in Cord Blood in Relation to Maternal Lifestyle as a Risk Factor

Variables	Responses	Number Examined (%)	Number Positive (%)	*p-value*
How often do you wash your hands	Regularly	44(24.4)	16(8.9)	
	Occasionally	44(24.4)	16(8.9)	<0.001
	Rarely	92(51.2)	34(18.9)	
Do you wash your fruits and vegetables before eating	Regularly	14(7.8)	5(2.8)	
	Occasionally	48(26.7)	18(10.0)	<0.001
	Rarely	118(65.5)	43(23.9)	
How often do you treat your drinking water	Regularly	8(4.4)	3(1.6)	
	Occasionally	62(34.5)	22(12.2)	<0.001
	Rarely	110(61.1)	41(22.8)	
How often do you eat partially cooked meat	Regularly	109(60.6)	40(22.2)	
	Occasionally	35(19.4)	13(7.2)	<0.001
	Rarely	36(20.0)	13(7.2)	
Do you own or have contact with pet(s)	Regularly	55(30.6)	20(11.1)	
	Occasionally	62(34.4)	23(12.8)	<0.001
	Rarely	63(35.0)	23(12.8)	
TOTAL		180	66(36.7)	

## Discussion

Toxoplasmosis is one of the most prevalent congenital infections and poses serious implications for pregnant women, fetuses, and newborns. In this study, the prevalence of toxoplasmosis and its association with maternal factors (age, educational status, and occupation) and child factors (sex and birth weight) were evaluated. The overall prevalence of toxoplasmosis observed was 36.7%, indicating a substantial burden within the studied population. This high rate increases the risk of congenital toxoplasmosis, which can lead to adverse neonatal outcomes such as neurological deficits and ocular diseases, potentially imposing additional demands on healthcare resources, including the need for specialized diagnostic and therapeutic services.

The elevated prevalence observed in this study may be attributed to a general lack of awareness about the infection and the fact that testing is not routinely conducted in the study area. A similar study in Ghana reported a prevalence of 29.2% (12). In studies conducted in the United States, the seroprevalence of toxoplasmosis in cord blood varied geographically—from 17.5% in the West to 20.5% and 29.2% in the South-Midwest and Northeast, respectively (13). These variations may be due to differences in climatic conditions, standards of living, and awareness levels regarding the infection. On the contrary, some scholars conducting related studies in Ghana reported even higher prevalence rates of 44.9% (14) and 73.6% (15). These differences could be explained by geographical variation, differences in diagnostic methods, or poor hygienic practices.

This study found no statistically significant association between maternal age and toxoplasmosis infection in cord blood. These findings are consistent with the results of other scholars (14,15,16), who also reported no significant relationship between maternal age and T. gondii infection. However, some related studies noted an increased prevalence of T. gondii infections in older age groups among females (5,13).

Although infection prevalence in relation to maternal education level was statistically insignificant in this study, the highest prevalence was found among mothers with only a basic level of education (up to secondary school). This may be due to limited knowledge about the disease, potentially leading to greater exposure to environmental contamination. Similar observations were made by other researchers in related studies (4,5,15).

Self-employed mothers had a higher prevalence of toxoplasmosis (17.2%) in this study. This finding aligns with related studies where self-employed women, particularly those in the food market, had a higher prevalence of the infection (4,5,14).

Male neonates had a higher prevalence of toxoplasmosis (13.9%) compared to females (7.2%), although this difference was statistically insignificant. A similar pattern was reported in another study (16,17). Karel (2022) reported that low birth weight was associated with newborns who tested positive for congenital toxoplasmosis. This observation aligns with the findings of the current study, where the prevalence of toxoplasmosis was associated with lower birth weight, suggesting that toxoplasmosis in cord blood may increase as neonatal weight decreases (17).

Maternal factors such as lack of awareness, consumption of undercooked meat, contact with pets, consumption of unwashed vegetables, and poor hand hygiene were identified as significant risk factors for increased seroprevalence of Toxoplasma gondii infection in this study. Several other studies have similarly highlighted these risk factors as significantly contributing to the increased prevalence of T. gondii infection (3,9,18,19,20).

In conclusion, the prevalence of toxoplasmosis in cord blood was relatively high and significant in this study. It was found to be associated with maternal educational status and the birth weight of the child. Therefore, we recommend the implementation of routine serological screening for T. gondii infection among pregnant women to facilitate early detection and intervention, thereby reducing the rate of vertical transmission. Targeted educational campaigns should be launched to raise awareness about transmission routes, preventive measures, and the importance of early diagnosis among women of childbearing age. Promoting practices such as proper food handling, consumption of well-cooked meats, avoidance of contact with cat feces, and maintaining proper hand hygiene—especially during pregnancy and when handling raw meat—should be strongly encouraged to mitigate infection risk.

We acknowledge that this study was limited to a single healthcare facility, which may affect the generalizability of the findings to a broader population. The relatively small sample size may have reduced the statistical power to detect significant associations between T. gondii infection and some risk factors. Additionally, we were unable to follow up on IgG antibody titers for at least two months to confirm congenital toxoplasmosis. The exclusive use of serological diagnosis, without molecular techniques, could have led to an underestimation of T. gondii prevalence.

Future research should focus on conducting multicenter studies across diverse geographical regions to enhance external validity and provide a more comprehensive understanding of toxoplasmosis prevalence in cord blood in relation to maternal factors. Longitudinal cohort studies should be implemented to monitor infection dynamics over time and establish causal relationships between various risk factors and T. gondii infection. Furthermore, evaluating the effectiveness of preventive strategies—such as educational interventions and screening programs—will be crucial in reducing both the prevalence and adverse outcomes of toxoplasmosis.
